# A tale with a Twist: a developmental gene with potential relevance for metabolic dysfunction and inflammation in adipose tissue

**DOI:** 10.3389/fendo.2012.00108

**Published:** 2012-08-30

**Authors:** Anca D. Dobrian

**Affiliations:** Department of Physiological Sciences, Eastern Virginia Medical SchoolNorfolk, VA, USA

**Keywords:** Twist1, human obesity, T cells, inflammation, visceral fat, IL12, type 2 diabetes

## Abstract

The Twist proteins (Twist-1 and -2) are highly conserved developmental proteins with key roles for the transcriptional regulation in mesenchymal cell lineages. They belong to the super-family of bHLH proteins and exhibit bi-functional roles as both activators and repressors of gene transcription. The Twist proteins are expressed at low levels in adult tissues but may become abundantly re-expressed in cells undergoing malignant transformation. This observation prompted extensive research on the roles of Twist proteins in cancer progression and metastasis. Very recent studies indicate a novel role for Twist-1 as a potential regulator of adipose tissue (AT) remodeling and inflammation. Several studies suggested that developmental genes are important determinants of obesity, fat distribution and remodeling capacity of different adipose depots. Twist-1 is abundantly and selectively expressed in the adult AT and its constitutive expression is significantly higher in subcutaneous (SAT) vs. visceral (VAT) fat in both mice and humans. Moreover, Twist1 expression is strongly correlated with BMI and insulin resistance in humans. However, the functional roles and transcriptional downstream targets of Twist1 in AT are largely unexplored. The purpose of this review is to highlight the major findings related to Twist1 expression in different fat depots and cellular components of AT and to discuss the potential mechanisms suggesting a role for Twist1 in AT metabolism, inflammation and remodeling.

## Regulation of gene transcription by Twist1

**Figure 1:**
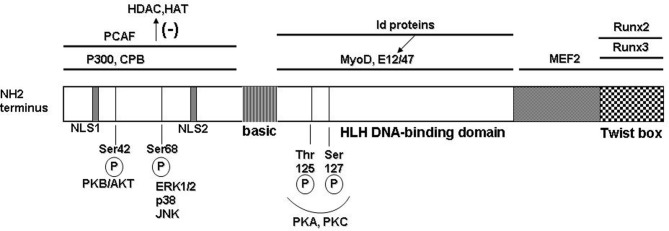
**Molecular structure of the human Twist protein.** Twist1 has the typical modular structure of the basic helix-loop-helix (bHLH) transcription factor family. At the N-terminus region 2 nuclear localization signals (NLS) are located at positions 37–40 and 73–77, respectively. The protein–protein interactions are delineated with black bars on top; PCAF = p300/CBP associated factor; CPB = cAMP response element binding protein; binding of the partner proteins in this region inhibits recruitment of the histone remodeling proteins HDAC (histone de-acetylase) and HAT (histone acetyl transferase). Twist1 can form heterodimers with other bHLH transcription factors that subsequently bind to the consensus E-box sequences in target genes. Some of the binding partners are the universal E-box proteins E12 and E47; also, Myo D involved in muscle differentiation; Id family of proteins can sequester the E-box transcription factors and therefore prevent formation of the dimers with Twist1. In the C-terminal region, the functional Twist box domain can bind Runx 2 or 3 (runt transcription factors). A number of functional Ser/Thr residues allow post-translational modification of Twist1 (illustrated on the bottom).

The transcriptional regulatory outcomes of Twist1 are dictated by several factors that include spatial and temporal cellular expression, protein–protein interactions, binding partner specificity, and post-transcriptional regulation by phosphorylation (Figure 1).

Twist1 has the generic modular structure of the family of basic helix-loop-helix (bHLH) transcription factors with an N-terminus region responsible for protein–protein interactions; a basic HLH domain that is capable to form homo- or heterodimers with other E-box proteins which upon dimerization recognize and bind a canonical consensus sequence in the DNA; and, a C-terminal region that contains the Twist box domain characterized both as an activation and as a repressor domain (Thisse et al., 1987, 1988; Murre et al., 1989; Kadesch, 1993) (Figure 1). The accessibility and choice of the binding partners is dictated by several mechanisms including phosphorylation state of Twist1 and accessibility of the other E-box proteins (Connerney et al., 2006; Firulli and Conway, 2008). For example, the id class of bHLH transcription factors can sequester binding partners for Twist, such as E12, therefore impacting on the type of dimer and transcriptional outcome (Benezra et al., 1990; Connerney et al., 2006). Partner choice can also be dictated by the level of Twist1 phosphorylation. PKA, PKC, PKB/Akt, and PP2 can all determine phosphorylation levels of Twist1 at highly conserved Ser and Thr residues in the HLH region (Firulli et al., 2003; Firulli and Conway, 2008; Vichalkovski et al., 2010; Hong et al., 2011). Also, the redox state of the cell can determine the transcriptional outcome by formation of homodimers that are stabilized by disulfide bonds therefore preventing formation of heterodimeric complexes with other E-box proteins (Danciu and Whitman, 2010).

Twist1 can also influence gene expression via epigenetic mechanisms. Interaction of Twist1 with HATs and HDACs, for example HDAC4, induces recruitment of the latter at the cis-regulatory regions in DNA resulting in histone modification and repression of gene transcription (Hamamori et al., 1999; Gong and Li, 2002). Also, a recent paper showed that Twist1 directly regulates formation of miRNA such as *let-7i* which results in downstream activation of Rac1 (Yang et al., 2012). All of these potential mechanisms contribute to the transcriptional outcome and depend upon the cell type and microenvironment.

Twist2 is the other member of the *twist* subfamily and presents 66% homology with Twist1 with over 98% homology in the basic and HLH domains of the protein (Franco et al., 2010). The common intron/exon organization of the two genes likely reflects an evolutionary gene duplication event. Although, some of the actions of Twist1 and 2 are redundant, specific tissue and temporal expressional patterns suggest also separate roles and mechanisms on gene regulation (Franco et al., 2010).

While the Twist1 related mechanisms involved in the mesoderm specification and the dorsal–ventral patterning during early embryo development were extensively studied, the role of Twist1 in adult tissues is only beginning to be understood. The most studied mechanisms are related to contribution of Twist1 to tumorigenesis and invasiveness in epithelial cancers (Yang et al., 2004; Kwok et al., 2005; Lee et al., 2006; Puisieux et al., 2006). Also, several papers evidenced the role of Twist1 in chronic inflammation and immunity (Sosic et al., 2003; Sharabi et al., 2008; Doreau et al., 2009). The recent findings showing distinct pattern of expression of Twist1 in various fat depots in mice and humans (Pan et al., 2009; Pettersson et al., 2010a) as well as correlation of Twist1 expression with BMI and insulin resistance and AT inflammation (Pettersson et al., 2010b) are granting future efforts towards understanding the specific Twist1 targets and mechanisms employed for translational regulation in AT as well as the functional relevance for pathologies such as insulin resistance, type 2 diabetes and vascular disease.

Twist2 is also expressed in AT in mice and humans (Pan et al., 2009; Pettersson et al., 2010b), however no depot-related expressional difference was reported or any correlation with obesity or insulin resistance (Pettersson et al., 2010b). Moreover, Twist1 is selectively expressed in brown and white fat, while Twist2 has a more ubiquitous expressional pattern (Pan et al., 2009; Pettersson et al., 2010a). Therefore, in the remaining of the review the focus will be on the evidence for presence and roles of Twist1 in AT.

## Twist1 expression and functional roles in adipose tissue

AT is an endocrine organ characterized by significant plasticity and cellular heterogeneity. Twist1 expression was reported for both the white (Pettersson et al., 2010b) (WAT) and brown (Pan et al., 2009) (BAT) ATs. Moreover, Twist1 was selectively expressed in AT typically at levels over 10-fold higher than in other tissues in adult rodents. Of the cells component of AT, white and brown adipocytes express abundantly Twist1 (Pan et al., 2009; Pettersson et al., 2010a). Also, endothelial cells, macrophages, and T cells in WAT express Twist1 although mRNA expression is 2–3-fold lower compared to adipocytes (Pettersson et al., 2010a). In addition, Twist1 expression increases by 2-fold in pre-adipocytes after 8 days of differentiation in culture conditions. Like other developmental genes such as HoxA5, Tbx15, and Gpc4, Twist1 shows distinct pattern of expression in different adipose depots. Expression of Twist1 is 6–8-fold higher in the subcutaneous (SAT) vs. visceral (VAT) human AT (Pettersson et al., 2010b). The reason for this expressional difference between the depots is not known but suggests that Twist1 plays potentially important roles in adipocyte development and fat distribution.

The unique developmental gene expression signature of the different fat depots is largely independent of nutritional state, at least in rodent models of obesity. Nonetheless, Twist1 gene expression is highly correlated with BMI and is reduced in obesity and increased following surgical or caloric restriction weight loss in both SAT and VAT in humans (Pettersson et al., 2010b). Also, Twist1 expression is negatively correlated with HOMA-IR and adipocyte size in humans (Pettersson et al., 2010b). This observation does not directly support a causative relationship between Twist1 and obesity and insulin resistance however strongly suggests contribution of this gene to the pathogenesis of these metabolic disorders.

In the following chapters the potential functional roles of Twist1 in AT and contributing mechanisms to the pathogenesis of obesity and insulin resistance will be discussed.

### Twist1 and WAT remodeling in obesity

AT is subjected to active remodeling throughout adult life. Increased caloric intake leads to expansion of the tissue by up to 30-fold contributed by both the adipocyte number and hypertrophic remodeling (Sun et al., 2011). Conversely, with weight loss, the size of the cells is reduced and pro-apoptotic mechanisms and reduced adipocyte differentiation match the newly reduced demand for substrate storage (Sethi and Vidal-Puig, 2007). The capacity of AT to expand in response to excessive caloric intake is depot specific and subject to inter-individual variation. It is believed that 25–70% of the observed variability in BMI and adipocyte number is genetically determined however the contributing genes are not known (Nelson et al., 2000; Baker et al., 2005).

The Twist1 gene is highly expressed in SAT vs. VAT and is correlated with BMI (Pettersson et al., 2010b). Gene and protein expression is significantly decreased in both SAT and VAT depots with obesity and increases with both surgical and caloric restriction weight loss in humans (Pettersson et al., 2010b). Interestingly, although Twist2 is also expressed in WAT there is neither depot specific difference in the expression of the latter nor any correlation with BMI (Pettersson et al., 2010b). This suggests a potentially important role for Twist1, but not as much for Twist2 in AT remodeling in adult life. Studies from Twist1 knockout mutants indicate that while the Twist1 homozygotes are embryonically lethal due to increased apoptosis in multiple tissues, the Twist2^−/−^ homozygotes as well as Twist1^+/−^/Twist2^+/−^ compound heterozygote mice die within 7–15 days after birth due to severe muscle wasting and systemic inflammation (Sosic et al., 2003). Interestingly, the latter mutants are born with no SAT and severely reduced VAT which explains the lethal cachexia. This result suggests that Twist1 and 2 act redundantly to control the formation of AT during embryogenesis. However, recent data from Pan et al. demonstrate that Twist1 is not a contributor to adipogenesis in either WAT or BAT in adult animals (Pan et al., 2009).

AT remodeling in response to excess caloric intake involves both hyperplastic and hypertrophic changes in the adipocytes. These processes require both cell migration and extracellular matrix remodeling. It is known that Twist1 plays an important role in epithelial mesenchymal transition (EMT) in various forms of malignant tumors and high levels of Twist1 are positively correlated with the metastatic potential (Martin and Cano, 2010; Qin et al., 2012). Twist1 contributes to EMT by regulating pathways involved in cell migration, cadherin E and N expression, and extracellular matrix remodeling, in particular of collagen (Martin and Cano, 2010). Therefore, it is tempting to speculate that Twist1 may play a role in cell migration and/or extracellular matrix re-organization both of which are associated with active AT remodeling (Lee et al., 2010). Interestingly, some evidence points out towards the SAT as the first depot to respond to high caloric intake by an increase in cell size and number (Arner et al., 2010; Hoffstedt et al., 2010; Kursawe et al., 2010). Apparently the adipocyte precursors are more readily mobilized in the SAT and this may be controlled in part by the higher levels of Twist1 expression of SAT pre-adipocytes and adipocytes. However, direct involvement of Twist1 in any of these processes is missing.

Also, the transcriptional mechanisms and Twist1 target genes in different adipose depots are largely unknown. One recently described mechanism by which Twist1 contributes to migration of malignant cells is via downregulation of the miRNA *let7i* (Yang et al., 2012). Interestingly, the *let7* family of miRNAs, including *let7i* was reportedly decreased in AT and adipocytes in obese vs. lean humans (Arner et al., 2012). Whether, this is one of the mechanisms by which Twist1 contributes to cell migration in AT remains to be established. Also, expressional changes in several collagen genes were reported as the second most prominent change in SAT following profound fat loss in humans (Dankel et al., 2010). In a rodent model of proliferative valvular heart disease, that over-expresses Twist1, several fibrillar collagens were dysregulated and collagen 2a1 was identified as a direct transcriptional target of Twist1 (Chakraborty et al., 2011). Therefore, the direct role of Twist1 in matrix remodeling that accompanies AT expansion in obesity is conceivable and is worth future investigation.

### Roles of Twist1 in adipocyte metabolism and potential relevance for insulin resistance

**Figure 2:**
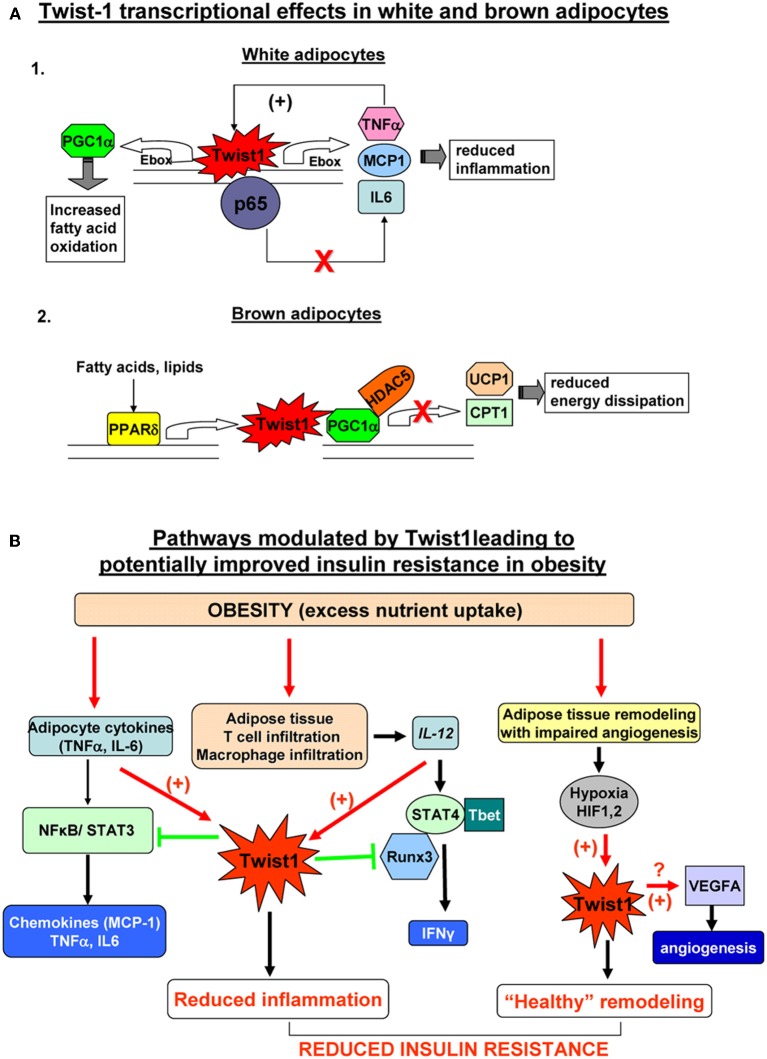
**(A)** Twist1 transcriptional effects in white (Thisse et al., 1987) and brown (Thisse et al., 1988) adipocytes. 1. In white human adipocytes Twist1 participates in basic transcription of TNFα and IL6, possibly by direct binding to their Ebox consensus sequences; however, increased formation of TNFα, up-regulates Twist which in turn will bind to p65 and prevent trans-activation of the TNFα and IL6 genes by the active NFkB complex; also, Twist increase expression attenuates the effect of TNFα on MCP-1 production; by participating in this negative feedback loop, Twist1 may limit inflammation in white adipocytes; Twis-1 also has metabolic effects by direct binding to PGC1α (PPARγ co-activator 1) which in turn increases fatty acid oxidation. 2. In mouse brown adipocytes Twist1 is up-regulated by PPARδ ; Twist1 binding to PGC1α promotes recruitment of HDAC5 at the promoter of the target genes UCP1 (uncoupling protein 1) and CPT1 (carnitine palmitolyl transferase 1) which results in reduced brown fat energy dissipation. **(B)** Pathways modulated by Twist1 leading to potentially improved insulin resistance in obesity. Excess nutrient excess and obesity leads to adipocyte inflammation, immune cell recruitment in adipose tissue and hypertrophic and hyperplastic remodeling of AT. Inflamed adipocytes secrete pro-inflammatory cytokines (TNFα, IL6, etc.) which via NFkB and STAT3 lead to formation of chemokines including MCP1 (macrophage attractant protein 1) that further exacerbate tissue inflammation. Twist1 is activated by TNFα and acts via a negative regulatory loop at the level of NKkB to limit formation of pro-inflammatory cytokines and chemokines; the macrophages infiltrating adipose tissue in obesity are a rich source of IL12 which induces Th1 polarization of T cells and via STAT4 and Tbet induces formation of IFNγ ; Twist1 is also up-regulated by IL12/STAT4; Twist may bind to Runx3 and prevent the latter to interact with Tbet therefore reducing IFNγ transcription; by limiting the pro-inflammatory effects of IL12, Twist1 further prevents local inflammation; active expansion of adipose tissue in obesity may not be matched by adequate angiogenesis; local hypoxia activates HIF1α and HIF2α (hypoxia-inducible factors) which can directly bind and up-regulate Twist1 expression; Twist1 in turn may promote formation of VEGFA that induces angiogenesis and restores the adequate oxygen and nutrient demand of the adipose tissue leading to “healthy” remodeling. Collectively, these pathways modulated by Twist1 may lead to prevention of insulin resistance in obesity.

Some direct and indirect recent evidence support a role for Twist1 in regulation of metabolism in the white and brown adipocytes. In brown adipocytes Twist1 is a key orchestrator of PGC-1α transcriptional regulation on target genes involved in uncoupling and mitochondrial biogenesis (Pan et al., 2009). In an elegant study, Pan et al. showed that PGC-1α physically interacts with Twist1 at the N-terminal domain of the latter. This interaction takes place at the promoter site of PGC1α target genes and results in recruitment of HDAC5 which in turn inhibits gene expression of UCP1 and mCPT1. Also, PPARδ induces Twist1 expression upon ligand activation but does not affect the basal Twist1 expression (Pan et al., 2009). Therefore, it appears that Twist1 serves as a negative feedback regulatory loop to finely tune PGC1α/PPARδ controlled brown fat metabolism in response to nutrient status and to therefore ensure energy homeostasis (Figure 2A). Since a decrease of energy expenditure was proposed to contribute to white fat accumulation and obesity this study suggests that Twist1 may be a positive indirect contributor to fat accumulation leading to obesity. However, additional studies in human brown adipocytes are critical to confirm this mechanism described in rodents and to establish the potential contribution of Twist1 to regulation of energy expenditure and obesity in humans.

In white human adipocytes, Twist1 also regulates fatty acid oxidation (Pettersson et al., 2010a). Silencing of Twist1 using siRNA determined decreased PGC1α and CPT1 expression along with reduced fatty acid oxidation and no changes in genes controlling basal lipolysis (Pettersson et al., 2010a) (Figure 2A). This result suggests that Twist1 is a promoter of fatty acid oxidation in white adipocytes arguing for a potentially beneficial metabolic effect. The detailed transcriptional mechanisms were not described in the white adipocytes. It is possible that other bHLH transcription factors present in adipocytes, such as the id class of proteins by binding to E-box proteins such as, E12 or E47, sequester the latter therefore preventing them to heterodimerize with Twist1. In effect, Twist1 may form preferentially homodimers which may have opposite transcriptional effects on target genes compared to Twist1/E-box heterodimers. An interesting observation is that both id1 and id3 knockout mice are protected from obesity induced insulin resistance and AT inflammation (Akerfeldt and Laybutt, 2011; Cutchins et al., 2011; Satyanarayana et al., 2012). One may therefore speculate that lack of id3 proteins could facilitate formation of Twist1 heterodimers with E-box partners and exert a positive effect on AT insulin sensitivity.

### Twist1 as potential regulator of inflammation in adipose tissue

Ample evidence suggests that VAT inflammation provides a mechanistic link between obesity and insulin resistance (de Luca and Olefsky, 2008). Preferential immune infiltration and increased pro-inflammatory cytokine production by both the adipocytes and granulocytes/lymphocytes in VAT compared to SAT was described in both murine models and in human obesity (Dobrian et al., 2010; Lumeng and Saltiel, 2011; Morris et al., 2011). The reasons for depot-specific differences in inflammation are incompletely understood. Since Twist1 is differentially expressed in the VAT and SAT and inversely correlated with BMI it is possible that Twist1 may have anti-inflammatory effects that are more prominent in SAT compared to VAT and may account in part for the differences in inflammation between the two depots.

Twist1 is expressed in T cells, macrophages, dendritic cells, and natural killer cells (Franco et al., 2010). Twist1 and 2 proteins were found to perform important roles in lymphocyte function and maturation. In Th1 cells Twist1 is induced by NFkB and as a result of IL12/STAT4 signaling (Sosic et al., 2003; Sharif et al., 2006). Twist1 induced expression in turn reduces production of IFNγ and TNFα, therefore, preventing their pro-inflammatory action in the context of chronic inflammation (Niesner et al., 2008). Similarly, in macrophages, IFNγ induces Twist1 expression which, in turn, reduces TNFα production (Sharif et al., 2006). The reduction of pro-inflammatory cytokine production is achieved via a negative feedback regulatory loop at the level of NFkB. It was demonstrated that Twist1 and 2 both physically interact with NFkB, in an evolutionary conserved fashion, leading to inhibition of p65 and repression of NFkB transactivation of cytokines gene promoters (Sosic et al., 2003). The importance of this negative feedback loop was demonstrated by the phenotype of *twist1*^+/−^/*twist2*^+/−^ mice that die perinataly due to severe systemic inflammation and cachexia. A recent paper elegantly showed that in Th1 cells, Twist1 negatively regulates expression and function of key transcription factors such as, T-bet, STAT4, and Runx3 (Pham et al., 2012). One of the mechanisms by which Twist1 limits IFNγ production in Th1 cells is by physical interaction with Runx3 that prevents association of the latter with T-bet at the *Ifng* locus (Pham et al., 2012). These seemingly common mechanisms of action of Twist1 in various immune cells result in a reduction of pro-inflammatory cytokine production. Since all of these immune cells infiltrate AT in obesity and contribute to local inflammation selectively in the VAT, (Lumeng and Saltiel, 2011) it is conceivable to hypothesize that increased Twist1 expression in SAT contributes to limiting inflammation in this depot; a decrease of Twist1 expression in AT in obesity suggests that the pro-inflammatory cytokine feedback inhibition by Twist1 is impaired and may account for the exacerbation of inflammation (Figure 2B).

Twist1 also affects cytokine production in white adipocytes in humans (Pettersson et al., 2010a). Apparently, Twist1 is required for the basal transcription of IL6, TNFα, and MCP-1 possibly via direct interaction, as all of these genes contain E-boxes in their gene promoters (Pettersson et al., 2010a). However, in a pro-inflammatory setting, when TNFα is increased, the presence of Twist de-sensitizes the effects of TNFα on MCP1 mRNA expression and secretion (Pettersson et al., 2010b) (Figure 2A). This is achieved in part by a reduction of at least two important components in the TNFα signaling pathway: *RelA* and *TNF-R1* (Pettersson et al., 2010b). This suggests that Twist1 could have different effects in AT depending upon the local *milieu*. Interestingly, Twist1 expression is also inversely associated with adipocyte size and HOMA-IR in obese humans suggesting a possible role in insulin sensitivity (Pettersson et al., 2010b). Adiponectin is a cytokine produced exclusively in AT with anti-inflammatory and insulin sensitizing roles (Turer and Scherer, 2012). Adiponectin expression correlated strongly and positively with Twist1 expression in SAT of obese subjects (Pettersson et al., 2010b). However, in isolated adipocytes, Twist1 silencing did not affect adiponectin levels (Pettersson et al., 2010a). This finding suggests that is unlikely that there might be a direct link between the two proteins. One can speculate that an indirect link may be via the id3/E47 interaction. Adiponectin gene transcription can be repressed by id3 which interacts with E47 thereby preventing SREBP-1c mediated trans-activation of the adiponectin gene (Doran et al., 2008). One possible explanation is that in inflammatory conditions when id3 expression is increased the latter may exert an inhibitory effect on adiponectin transcription and, conversely high levels of Twist1 may outcompete id3 in interaction with E47 and therefore could limit the id3 inhibitory effect. Additional studies are key to understand the mechanisms involved in regulation of adiponectin transcription by Twist1.

Collectively, this data suggests that Twist1 could limit inflammation in AT via effects on adipocytes and on the immune cells infiltrating the AT (Figure 2B).

### Regulation of Twist1 by hypoxia and implications for angiogenesis in adipose tissue remodeling in obesity

The unique plasticity of AT in response to caloric intake ensures proper energy homeostasis. Expansion of AT requires adequate recruitment of adipocytes, and matching nutrient and O_2_ demands provided by adequate vasculature (Rutkowski et al., 2009). The failure to mount an adequate hyperplastic and angiogenic response leads to adipocyte hypertrophy, hypoxia and subsequent inflammation, and fibrosis that may ultimately contribute to the onset of insulin resistance (Hosogai et al., 2007; Pasarica et al., 2009; Spencer et al., 2011). Numerous evidence shows that in most but not in all cases obesity is accompanied by the “unhealthy” remodeling of AT (Sun et al., 2011). One of the major contributors is impaired angiogenesis, with the subsequent hypoxia, inflammation and fibrosis.

Twist1 is regulated at transcriptional level by both HIF1α and HIF2α in response to a hypoxic stimulus. HIF1α regulates Twist1 by direct binding to the hypoxia-response element in its promoter (Yang et al., 2008) while HIF2α binds at the level of intronic hypoxia response elements in the Twist1 gene (Gort et al., 2008). Up-regulation of Twist1 by the HIF proteins in highly hypoxic tumors is considered key for the EMT and tumor metastasis (Yang et al., 2008). Also, Twist1 was showed to induce angiogenesis in hepatocellular carcinomas (Niu et al., 2007) and was correlated with VEGF expression in breast cancer tumors (Mironchik et al., 2005). There is no evidence for a direct angiogenic role of Twist1 in AT in response to hypoxia or for the downstream molecular targets, but it is tempting to propose that high levels of Twist1 expression may promote angiogenesis in a fast growing AT therefore contributing to healthy remodeling of AT in obesity (Figure 2B).

## Conclusion

Recent findings showed unexpected and complex roles for developmental transcription factors in adult tissues undergoing active remodeling, such as, solid tumors and AT and relationships with pathogenesis of cancer and metabolic diseases. The potential involvement of Twist1 in AT metabolism, inflammation, and remodeling grants future research to unravel the molecular targets and transcriptional programs involved. Understanding the complex regulation of Twist1 in cells component of AT ultimately can generate new therapies to control AT healthy remodeling in response to nutritional challenges.

### Conflict of interest statement

The author declares that the research was conducted in the absence of any commercial or financial relationships that could be construed as a potential conflict of interest.
